# Psychological and contextual determinants of public CPR intention in Hubei Province: an extended theory of planned behavior study

**DOI:** 10.3389/fpubh.2026.1851252

**Published:** 2026-08-11

**Authors:** Kaiqi Chen, Ruijing Xu, Huiwen Wang

**Affiliations:** Union Hospital, Tongji Medical College, Huazhong University of Science and Technology, Wuhan, China

**Keywords:** bystander rescue, CPR, extended theory of planned behavior, first-aid training, Hubei, structural equation modeling

## Abstract

**Objective:**

This study examined psychological and contextual determinants of public willingness to perform cardiopulmonary resuscitation (CPR) in Hubei Province using an extended theory of planned behavior (ETPB) framework and explored training-associated changes after standardized first-aid training.

**Methods:**

A theory-driven cross-sectional survey with supplementary within-group pre-post training evaluation was conducted. Adults in Hubei Province were recruited between July 2022 and March 2023. The ETPB model included behavioral attitude, subjective norm, perceived behavioral control, implementation context, and facilitating conditions as predictors of CPR rescue intention. Confirmatory factor analysis and structural equation modeling were used to evaluate the theory-driven model. A supplementary exploratory multi-group SEM and path-by-path critical-ratio comparison were conducted to examine whether previous CPR/AED training experience moderated specific ETPB pathways. Training-associated changes were assessed among participants attending an 8-h Hubei Red Cross first-aider program.

**Results:**

A total of 2,156 valid questionnaires were analyzed. The ETPB scale showed acceptable preliminary reliability and validity. The structural model demonstrated acceptable fit and identified facilitating conditions and perceived behavioral control as the strongest predictors of CPR rescue intention, followed by subjective norm and behavioral attitude; implementation context was not significant. In the supplementary training evaluation, 652 participants completed paired assessments. Post-training improvements were observed in CPR/AED knowledge, practical performance, confidence, AED-use willingness, and ETPB domain scores related to CPR rescue intention. The path-by-path critical-ratio comparison identified two moderated pathways: perceived behavioral control to CPR rescue intention was stronger among trained participants, whereas facilitating conditions to CPR rescue intention was stronger among untrained participants.

**Conclusion:**

Public CPR rescue intention in Hubei Province was shaped mainly by perceived competence and enabling conditions rather than by attitude alone. Previous CPR/AED training experience moderated two key ETPB pathways. CPR promotion strategies should therefore strengthen perceived competence and confidence among trained laypeople, while improving accessible training opportunities and supportive learning conditions among untrained laypeople.

## 1 Introduction

Out-of-hospital cardiac arrest (OHCA) remains a time-critical public health emergency. In China, the overall incidence of OHCA has been reported as approximately 97.1 per 100,000 person-years, showing an upward trend compared with data from 10 years earlier (1, 2). Earlier epidemiological estimates also suggested that sudden cardiac death occurs at a rate of 41.8 per 100,000 population in China, corresponding to more than 540,000 deaths annually (3). Despite this substantial disease burden, community response remains insufficient. National and regional data indicate that bystander CPR rates in China remain low, with some studies reporting rates around 4.5% and considerable regional variation (2, 4). Public-access defibrillation is even less common; a review of OHCA resuscitation in China reported no bystander defibrillation in the included studies, and regional data from Shanghai showed AED use before ambulance arrival of only 1.43% (5–8). These data suggest that improving public knowledge alone may be insufficient unless willingness, confidence, and enabling conditions for bystander CPR are also addressed.

Previous AED-focused findings from the broader Hubei survey project have described public AED knowledge, willingness to use public-access defibrillation, reasons for willingness or unwillingness, and demographic correlates of AED use willingness (6). Those findings identified important AED-related knowledge gaps and implementation barriers. However, the ETPB items reported in the present manuscript primarily measure CPR rescue intention rather than combined CPR/AED intention. Therefore, in response to the reviewers’ comments, the present manuscript was refocused on CPR rescue intention, while AED-related findings are retained only as contextual background and as part of the training evaluation.

The theory of planned behavior (TPB) explains behavioral intention through attitude, subjective norm, and perceived behavioral control. In the context of bystander CPR, however, intention may also depend on scenario-specific barriers and enabling resources, including legal concerns, fear of causing harm, disease exposure, scene safety, training availability, and access to supportive learning opportunities. Therefore, this study adopted an extended theory of planned behavior (ETPB) framework by adding implementation context and facilitating conditions to the classical TPB constructs (9, 10). The importance of lay responder experience, including previous CPR/AED training exposure, has also been emphasized in an American Heart Association scientific statement (11)^.^ Therefore, previous training experience should be considered not only as a demographic background variable, but also as a contextual factor that may influence perceived competence, confidence, and willingness to perform CPR.

The objectives of this study were: (1) to identify psychological and contextual determinants of CPR rescue intention among adults in Hubei Province using an ETPB framework; and (2) to explore whether standardized Red Cross first-aid training was associated with changes in CPR/AED knowledge, practical performance, confidence, willingness, and ETPB-related domains.

## 2 Materials and methods

### 2.1 Study design

This study used a theory-driven observational design with a supplementary pre-post training evaluation. The main component was a cross-sectional survey conducted among adults in Hubei Province to examine psychological and contextual determinants of CPR rescue intention based on the extended theory of planned behavior (ETPB). The ETPB framework included behavioral attitude, subjective norm, implementation context, perceived behavioral control, and facilitating conditions as predictors of CPR rescue intention.

Before the formal survey, the questionnaire was developed and refined through literature review, expert consultation, semi-structured interviews, focus-group discussion, and pilot testing. These procedures were used to ensure item relevance, comprehensibility, and contextual appropriateness, but the study was not designed as a fully integrated mixed-methods study. After the cross-sectional survey, a supplementary within-group pre-post evaluation was conducted among participants who voluntarily attended the standardized Hubei Red Cross first-aider training program. The training evaluation was used to describe changes in CPR/AED knowledge, practical performance, confidence, willingness, and ETPB-related domains after training.

### 2.2 Participants, setting, and sampling

For the cross-sectional survey, adults residing in Hubei Province were recruited between July 2022 and March 2023. The inclusion criteria were as follows: (1) aged 18 years or older; (2) having lived in Hubei Province for at least 6 months during the previous year; (3) able to read and understand the questionnaire independently; (4) having full capacity for civil conduct; and (5) willing to participate and provide informed consent. Individuals who were unable to understand the questionnaire or who had severe mental illness that precluded valid participation were excluded.

Participants were recruited from multiple prefecture-level cities in Hubei Province, including Wuhan, Shiyan, Xiangyang, Xiaogan, and Xianning. Both offline and online recruitment strategies were used. Offline recruitment was conducted in public places such as communities, schools, workplaces, and public service settings. Online recruitment was conducted through convenience and snowball sampling using electronic questionnaires. To reduce duplicate responses, each online device or account was allowed to submit the questionnaire only once, and questionnaires with obvious logical inconsistencies, extremely short completion time, or substantial missing information were excluded.

For the supplementary training evaluation, participants were selected from members of the public who had completed the baseline survey and voluntarily registered for the standardized Hubei Red Cross first-aider training program. Eligible applicants completed the Red Cross rescuer information form and were notified to attend training. Participants who completed both pre-training and post-training assessments were included in the paired analysis. Available records contained 652 paired pre-training and post-training assessments.

### 2.3 Questionnaire development and measures

The questionnaire consisted of four parts: demographic and background information, CPR/AED knowledge, CPR rescue intention and related reasons, and ETPB-based psychosocial constructs. Demographic and background variables included age, sex, educational level, place of residence, residential population density, medical-related occupation, co-residing with a family member aged 60 years or older, cardiovascular disease history among household members, and previous CPR/AED training experience.

CPR/AED knowledge was assessed using multiple-choice items. The AED knowledge section included 10 items, with each correct answer scored as 10 points. The CPR knowledge section included 19 items. To ensure consistency in reporting, both AED and CPR knowledge scores were converted to standardized percentage scores ranging from 0 to 100, and a score of 60 or higher was regarded as satisfactory. Respondents who reported complete unfamiliarity with CPR or AEDs skipped the subsequent knowledge questions. This skip logic was used to reduce respondent burden, but it may have introduced selection bias because knowledge scores were calculated only among respondents with at least some baseline familiarity.

CPR rescue intention was assessed using items related to willingness to perform CPR in an OHCA emergency. The items assessed willingness to perform CPR for a patient with cardiac arrest, for acquaintances, and for strangers. Reasons for willingness or unwillingness to provide rescue were assessed using structured response options derived from literature review, semi-structured interviews, focus-group discussion, and expert input. These options covered common facilitating and barrier factors, including willingness to save lives, perceived responsibility, lack of confidence, fear of incorrect operation, fear of causing harm, disease exposure concerns, and legal concerns.

The ETPB-based section measured psychosocial constructs related to CPR rescue intention. The scale included six domains: behavioral attitude, subjective norm, implementation context, perceived behavioral control, facilitating conditions, and behavioral intention. Behavioral attitude reflected respondents’ evaluation of the value and usefulness of CPR. Subjective norm reflected perceived expectations from family, friends, and society. Implementation context reflected perceived barriers in real emergency situations, such as possible disputes, disease exposure, or the presence of others at the scene. Perceived behavioral control reflected respondents’ confidence in calling for help, judging the need for CPR, and performing CPR-related actions. Facilitating conditions reflected perceived access to CPR learning opportunities and support. Behavioral intention reflected willingness to perform CPR in different rescue situations. All ETPB items were rated on a 5-point Likert scale from 1 (strongly disagree) to 5 (strongly agree), with higher scores indicating stronger agreement with the corresponding construct.

AED-related items were retained only in the knowledge and training evaluation sections because the ETPB intention items listed in this manuscript primarily measured CPR rescue intention rather than combined CPR/AED intention.

### 2.4 Primary and secondary outcomes

The primary outcome of the cross-sectional survey was CPR rescue intention. In the ETPB model, CPR rescue intention was treated as the endogenous latent variable and was measured using three items reflecting willingness to perform CPR in different rescue scenarios, including general cardiac arrest, acquaintances, and strangers. Higher scores indicated stronger CPR rescue intention.

The main explanatory variables were five ETPB constructs: behavioral attitude, subjective norm, implementation context, perceived behavioral control, and facilitating conditions. Behavioral attitude was measured by three items assessing perceived usefulness, practicality, and value of CPR. Subjective norm was measured by four items assessing perceived expectations from family, friends, and the general public. Implementation context was measured by three items reflecting possible barriers in real emergency settings, including concern about trouble or litigation, disease exposure, and the presence of others at the scene. Perceived behavioral control was measured by four items assessing perceived ability to call emergency services, follow brief CPR instructions, judge whether CPR is needed, and perform chest compressions. Facilitating conditions were measured by three items assessing perceived availability and accessibility of CPR learning opportunities through organizations or online resources. All ETPB items were scored on a 5-point Likert scale, with higher scores indicating higher levels of the corresponding construct.

The supplementary training evaluation assessed changes in CPR/AED knowledge, practical performance, confidence, willingness, and ETPB-related domains before and after the standardized Hubei Red Cross first-aider training. Practical performance was evaluated using a standardized checklist covering scene assessment and calling for help, chest compressions, airway opening, rescue breathing, reassessment, and compression-to-ventilation ratio. Confidence was assessed by participants’ self-rated confidence in judging breathing, performing chest compressions, and providing rescue breathing.

### 2.5 ETPB framework and hypotheses

The ETPB model treated behavioral attitude, subjective norm, implementation context, perceived behavioral control, and facilitating conditions as exogenous latent variables and CPR rescue intention as the endogenous latent variable. Five hypotheses were tested: H1, behavioral attitude positively predicts CPR rescue intention; H2, subjective norm positively predicts CPR rescue intention; H3, implementation context positively predicts CPR rescue intention; H4, perceived behavioral control positively predicts CPR rescue intention; and H5, facilitating conditions positively predict CPR rescue intention.

### 2.6 Supplementary training evaluation

The supplementary training evaluation was embedded in the 8-h Hubei Red Cross first-aider training program. Technical content included first-aid principles, basic anatomy, vital-sign observation, recognition of cardiac arrest, CPR steps and quality standards, airway opening, rescue breathing, AED operation, electrode pad placement, scene safety, post-shock CPR continuation, and simulated rescue scenarios. Training used a combination of theoretical teaching, instructor demonstration, small-group practice, supervised hands-on practice using manikins, and assessment.

Non-technical content was aligned with the ETPB domains and included the lifesaving value of rescue, social responsibility, legal and safety concerns, confidence building, repeated supervised practice, available training pathways, and community support resources.

Practical performance was assessed using a standardized CPR/AED operation checklist based on Hubei Red Cross first-aider certification criteria. The checklist covered scene assessment and calling for help, chest compressions, airway opening, rescue breathing, reassessment, and compression-to-ventilation ratio. Certified instructors used unified criteria for assessment. Inter-rater reliability was not recorded in the available dataset and is therefore acknowledged as a limitation.

Overall satisfaction with course content was not assessed in the available dataset; this outcome should be included in future course evaluations.

### 2.7 Statistical analysis

Descriptive statistics were used to summarize participant characteristics, CPR/AED knowledge, confidence, willingness, and ETPB domain scores. Continuous variables were presented as mean and standard deviation, and categorical variables were presented as frequency and percentage. Group comparisons were conducted using t tests, analysis of variance, chi-square tests, or non-parametric tests as appropriate. Confirmatory factor analysis was conducted to examine whether the observed items adequately represented the six predefined ETPB constructs. Internal consistency was assessed using Cronbach’s alpha. Composite reliability and average variance extracted were used to evaluate construct reliability and convergent validity. Model fit was evaluated using chi-square divided by degrees of freedom, goodness-of-fit index, adjusted goodness-of-fit index, comparative fit index, and root mean square error of approximation. Structural equation modeling with maximum likelihood estimation was used to test the hypothesized relationships between ETPB constructs and CPR rescue intention. The primary SEM tested the theory-driven pathways from behavioral attitude, subjective norm, implementation context, perceived behavioral control, and facilitating conditions to CPR rescue intention. Previous CPR/AED training experience was considered an important contextual variable because it may influence perceived competence, self-confidence, perceived behavioral control, and CPR rescue intention. To further explore whether the ETPB pathways differed according to training background, a supplementary exploratory multi-group SEM was conducted. Participants were divided into trained and untrained groups according to self-reported previous CPR/AED training experience. The same ETPB structural model was estimated in both groups. An unconstrained model was compared with a structural path constrained model, and differences between models were evaluated using the chi-square difference test. To identify which specific pathways were moderated by previous CPR/AED training experience, path-by-path critical-ratio comparisons were further conducted. An absolute critical-ratio value greater than 1.96 was considered to indicate a statistically significant between-group difference at the 0.05 level. Because the critical-ratio comparison was based on unstandardized parameter estimates, standardized path coefficients were reported together with the critical-ratio results to facilitate substantive interpretation.

### 2.8 Ethical considerations

This study was approved by the Medical Ethics Committee of Tongji Medical College, Huazhong University of Science and Technology (Approval No. 2022-S097). Written informed consent was obtained from all participants before data collection.

## 3 Results

### 3.1 Participant characteristics

A total of 2,218 questionnaires were distributed and 2,156 valid questionnaires were included, giving a valid response rate of 97.2%. The mean age was 31.15 years (range: 18–88 years). Women accounted for 59.0% of participants. The sample included relatively high proportions of participants with college-level education (65.4%), urban residence (69.9%), and medical-related occupations (39.1%). Previous CPR/AED training was reported by 26.3% of respondents.

Previous CPR/AED training was reported by 568 respondents (26.3%), indicating that most participants had not received formal CPR/AED training before the survey. This variable was therefore considered important when interpreting CPR rescue intention and the ETPB pathways (Table 1).

**Table 1:** Demographic and background characteristics of the study participants.

Characteristic	Value
Total valid questionnaires	2,156
Mean age	31.15 years (18–88)
Female	1,271 (59.0%)
College-level education	1,411 (65.4%)
Urban residence	1,506 (69.9%)
Medical-related occupation	842 (39.1%)
Co-residing family member aged ≥60 years	958 (44.4%)
Previous CPR/AED training	568 (26.3%)

### 3.2 Contextual CPR/AED knowledge and willingness findings

Because detailed AED-focused descriptive findings from the broader Hubei survey project have been reported in our previous publication (8), the present manuscript only briefly summarizes CPR/AED knowledge and willingness findings as contextual background for the ETPB model and training evaluation. In the previous AED-focused study, we reported public AED knowledge, willingness to use public-access defibrillation, reasons for willingness or unwillingness, and demographic correlates of AED use willingness in Hubei Province (8). Therefore, these descriptive AED-related results were not repeated in detail in the present manuscript.

In the current dataset, nearly half of respondents achieved satisfactory standardized CPR/AED knowledge scores. However, operational knowledge gaps remained, particularly regarding AED operation sequence, electrode pad placement, immediate response to absent breathing, timing of reassessment, and the optimal time to initiate CPR after collapse. Among respondents who completed the AED willingness item, 75.4% reported willingness to use an AED in an OHCA emergency. CPR willingness varied by relationship to the victim, with respondents showing greater willingness to perform CPR for acquaintances, relatives, or friends than for strangers. The most frequently reported reasons for willingness were the instinct to save lives and a sense of responsibility, whereas reasons for unwillingness were mainly legal concerns, fear of incorrect operation, and fear of causing harm. These findings provided contextual support for focusing the present analysis on confidence, perceived behavioral control, facilitating conditions, and other ETPB-related determinants of CPR rescue intention.

### 3.3 ETPB item descriptive statistics

Behavioral attitude had the highest domain score, while perceived behavioral control and facilitating conditions had lower scores, suggesting that competence and enabling resources may be more limiting than attitudes (Table 2).

**Table 2:** Descriptive statistics for the extended theory of planned behavior items related to CPR rescue intention.

Domain	Item	Content	Mean ± SD
Behavioral attitude	F11	CPR is useful for saving patients with cardiac arrest.	4.44 ± 0.68
Behavioral attitude	F12	Learning CPR is practical.	4.43 ± 0.70
Behavioral attitude	F13	I feel a sense of achievement when CPR saves lives.	4.26 ± 0.76
Subjective norm	F21	My family would expect me to perform CPR if needed.	4.26 ± 0.89
Subjective norm	F22	My friends would expect me to perform CPR if needed.	3.62 ± 1.06
Subjective norm	F23	People generally expect me to help in a first-aid emergency.	4.14 ± 0.99
Subjective norm	F24	Everyone has a responsibility to help in a first-aid emergency.	4.34 ± 0.86
Implementation context	F31	I would use my CPR/first-aid skills even if I might face trouble or litigation.	3.95 ± 0.96
Implementation context	F32	I would use my CPR/first-aid skills even if I might be exposed to disease.	3.85 ± 1.03
Implementation context	F33	I would use CPR/first-aid skills when others are present at the emergency scene.	4.09 ± 0.94
Perceived behavioral control	F41	I can effectively call emergency assistance in an emergency.	4.28 ± 0.89
Perceived behavioral control	F42	I can perform CPR with a brief instruction checklist.	3.60 ± 1.12
Perceived behavioral control	F43	I can determine whether a person needs CPR.	3.42 ± 1.18
Perceived behavioral control	F44	I can correctly perform chest compressions.	3.31 ± 1.27
Facilitating conditions	F51	There are many CPR training opportunities.	3.70 ± 1.04
Facilitating conditions	F52	It is easy for me to learn CPR through organizations.	3.46 ± 1.16
Facilitating conditions	F53	It is easy for me to learn CPR online.	3.82 ± 1.12
Behavioral intention	F61	I am willing to perform CPR for a patient with cardiac arrest.	3.51 ± 1.05
Behavioral intention	F62	I am willing to perform CPR for acquaintances.	3.58 ± 1.12
Behavioral intention	F63	I am willing to perform CPR for strangers.	3.46 ± 1.13

### 3.4 Confirmatory factor analysis of the ETPB measurement model

Confirmatory factor analysis was conducted to examine whether the observed items adequately represented the six predefined ETPB constructs. The six-factor measurement model included behavioral attitude, subjective norm, implementation context, perceived behavioral control, facilitating conditions, and CPR rescue intention. Standardized factor loadings ranged from 0.505 to 0.974. Composite reliability values ranged from 0.786 to 0.916, and AVE values ranged from 0.551 to 0.787, indicating acceptable reliability and convergent validity. The model fit indices were acceptable, with CMIN/DF = 2.833, GFI = 0.868, AGFI = 0.821, CFI = 0.936, and RMSEA = 0.080 (Table 3).

**Table 3:** Reliability and convergent validity of the extended theory of planned behavior measurement model.

Factor	Items	Cronbach alpha	Range of standardized loading	CR	AVE
Behavioral attitude	F11-F13	0.900	0.697–0.974	0.916	0.787
Subjective norm	F21-F24	0.866	0.682–0.876	0.879	0.646
Implementation context	F31-F33	0.835	0.692–0.857	0.842	0.642
Perceived behavioral control	F41-F44	0.841	0.505–0.869	0.859	0.613
Facilitating conditions	F51-F53	0.859	0.781–0.850	0.858	0.669
Behavioral intention	F61-F63	0.785	0.725–0.774	0.786	0.551

### 3.5 Structural equation model

**Figure 1:**
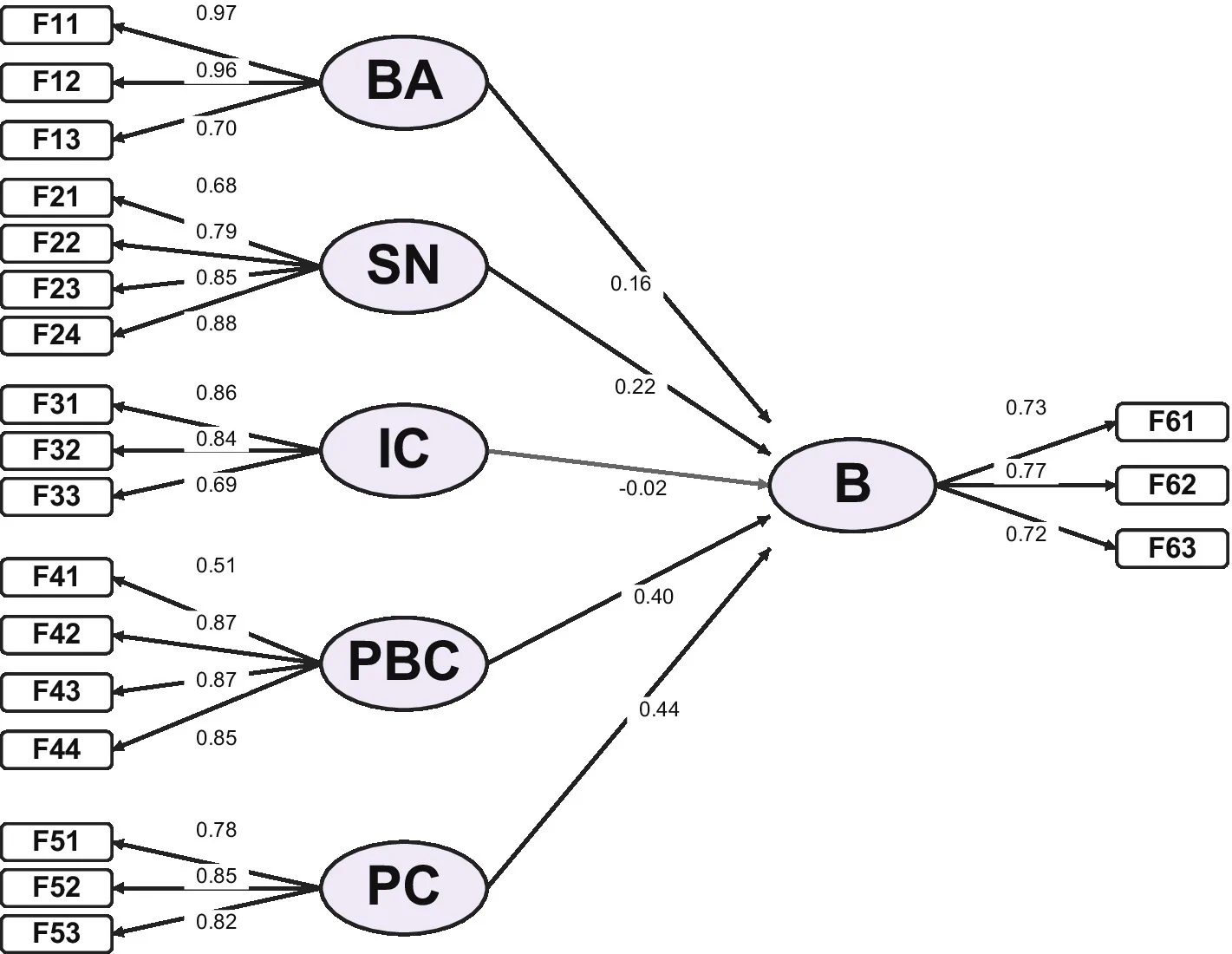
Path diagram of the original ETPB-based structural equation model of CPR rescue intention. Values indicate standardized factor loadings and standardized path coefficients.

In the primary ETPB structural model, behavioral attitude, subjective norm, perceived behavioral control, and facilitating conditions were significant positive predictors of CPR rescue intention, whereas implementation context was not statistically significant. Facilitating conditions and perceived behavioral control showed the strongest associations with CPR rescue intention. Because previous CPR/AED training experience may influence both perceived competence and rescue intention, it was further examined through supplementary multi-group SEM rather than being ignored as a background characteristic (Figure 1; Table 4).

**Table 4:** Standardized path coefficients and model-fit indices of the structural equation model predicting CPR rescue intention.

Path or index	Value	*p*	Interpretation
Behavioral attitude → rescue intention	0.159	*p* < 0.001	Significant
Subjective norm → rescue intention	0.223	*p* < 0.001	Significant
Implementation context → rescue intention	−0.020	*p* = 0.808	Not significant
Perceived behavioral control → rescue intention	0.397	*p* < 0.001	Strong predictor
Facilitating conditions → rescue intention	0.439	*p* < 0.001	Strongest predictor
CMIN/DF	2.833	—	Acceptable
GFI	0.868	—	Acceptable
AGFI	0.821	—	Acceptable
CFI	0.936	—	Good
RMSEA	0.080	—	Acceptable

### 3.6 Supplementary multi-group SEM by previous CPR/AED training experience

To further examine whether the ETPB model differed according to previous CPR/AED training experience, a supplementary exploratory multi-group SEM was conducted. Participants were divided into a trained group and an untrained group according to self-reported previous CPR/AED training experience. Among the 2,156 respondents, 568 had previously received CPR/AED training and 1,588 had not.

The unconstrained multi-group model showed good fit, suggesting that the ETPB framework could be applied to both trained and untrained participants. In both groups, perceived behavioral control and facilitating conditions remained the main predictors of CPR rescue intention. However, the relative strength of the standardized path coefficients differed descriptively between groups. In the trained group, perceived behavioral control appeared to show the strongest association with CPR rescue intention, followed by facilitating conditions and subjective norm. In the untrained group, facilitating conditions appeared to show the strongest association, followed by perceived behavioral control and subjective norm. Behavioral attitude was a significant but weaker predictor in both groups, whereas implementation context was not significant in either group (Table 5).

**Table 5:** Multi-group structural equation modeling results by previous CPR/AED training experience.

Path	Trained group β	*p*	Untrained group β	*p*
Behavioral attitude → CPR rescue intention	0.132	0.006	0.171	<0.001
Subjective norm → CPR rescue intention	0.205	<0.001	0.231	<0.001
Implementation context → CPR rescue intention	−0.018	0.741	−0.024	0.692
Perceived behavioral control → CPR rescue intention	0.462	<0.001	0.354	<0.001
Facilitating conditions → CPR rescue intention	0.361	<0.001	0.486	<0.001

The unconstrained model was then compared with a structural path constrained model in which the five structural paths from behavioral attitude, subjective norm, implementation context, perceived behavioral control, and facilitating conditions to CPR rescue intention were constrained to be equal across groups. The chi-square difference test suggested that constraining the structural paths to equality resulted in a statistically significant deterioration in model fit, indicating that previous CPR/AED training experience may moderate the overall pattern of ETPB pathways. Because this supplementary analysis did not include a full measurement invariance testing sequence, the multi-group and path-by-path critical-ratio results should still be interpreted cautiously (Table 6).

**Table 6:** Comparison of the unconstrained and structural-path-constrained multi-group models.

Model	χ^2^	df	CMIN/DF	CFI	RMSEA	Δχ^2^	Δdf	*p*
Unconstrained model	1084.326	382	2.838	0.931	0.078	—	—	—
Structural path constrained model	1101.842	387	2.847	0.928	0.079	17.516	5	0.004

The chi-square difference test indicated a significant difference between the unconstrained model and the structural path constrained model, suggesting that previous CPR/AED training experience may moderate the overall ETPB pathway pattern. This result supports the importance of considering training background when interpreting laypeople’s psychological and contextual determinants of CPR rescue intention (Table 7).

**Table 7:** Path-by-path critical-ratio comparison between participants with and without previous CPR/AED training experience.

Path	Trained group β	Untrained group *β*	Critical ratio	*p*	Moderated path
Behavioral attitude → CPR rescue intention	0.132	0.171	−1.42	0.156	No
Subjective norm → CPR rescue intention	0.205	0.231	−0.98	0.327	No
Implementation context → CPR rescue intention	−0.018	−0.024	0.21	0.834	No
Perceived behavioral control → CPR rescue intention	0.462	0.354	2.74	0.006	Yes; stronger in trained group
Facilitating conditions → CPR rescue intention	0.361	0.486	−3.12	0.002	Yes; stronger in untrained group

To identify the specific moderated pathways, path-by-path critical-ratio comparisons were further conducted. The results showed that the paths from perceived behavioral control to CPR rescue intention and from facilitating conditions to CPR rescue intention differed significantly between trained and untrained participants. Specifically, the association between perceived behavioral control and CPR rescue intention was stronger among trained participants than among untrained participants (critical ratio = 2.74, *p* = 0.006). In contrast, the association between facilitating conditions and CPR rescue intention was stronger among untrained participants than among trained participants (critical ratio = −3.12, *p* = 0.002). No significant between-group differences were observed for the paths from behavioral attitude, subjective norm, or implementation context to CPR rescue intention.

These findings indicate that previous CPR/AED training experience moderated two key ETPB pathways: the pathway from perceived behavioral control to CPR rescue intention and the pathway from facilitating conditions to CPR rescue intention. This suggests that trained individuals’ CPR rescue intention may depend more strongly on perceived competence and confidence, whereas untrained individuals’ intention may depend more strongly on external enabling conditions, such as accessible training opportunities and supportive learning resources.

### 3.7 Supplementary pre-post training evaluation

A total of 652 participants completed paired pre-training and post-training assessments. The proportion classified as having good overall knowledge increased from 60/652 (9.20%) before training to 314/652 (48.15%) after training (chi-square = 1160.0, *p* = 0.001). AED knowledge scores changed from 58.81 ± 26.79 to 86.29 ± 18.66 (t = 16.606, *p* = 0.001, Cohen’s dz = 0.650), and CPR knowledge scores changed from 60.93 ± 25.91 to 71.55 ± 12.82 (t = 7.203, p = 0.001, Cohen’s dz = 0.282).

Practical pass rates increased across all six assessed domains. Post-training pass rates were 95.2% for scene assessment and calling for help, 93.5% for chest compressions, 86.6% for airway opening, 99.6% for rescue breathing, 85.7% for reassessment, and 83.2% for compression-to-ventilation ratio. These findings should be interpreted as post-training changes because there was no control group (Table 8).

**Table 8:** Changes in CPR/AED knowledge, psychosocial constructs, and rescue willingness before and after first-aid training.

Outcome	Pre-training	Post-training	Test statistic	*p*
Overall good knowledge	60 (9.20%)	314 (48.15%)	χ2 = 1160.0	0.001
AED knowledge score	58.81 ± 26.79	86.29 ± 18.66	t = 16.606	0.001
CPR knowledge score	60.93 ± 25.91	71.55 ± 12.82	t = 7.203	0.001
Behavioral attitude	4.38 ± 1.13	4.47 ± 0.14	t = −4.436	0.016
Subjective norm	4.09 ± 0.32	4.23 ± 0.13	t = −7.045	0.002
Perceived behavioral control	3.65 ± 0.44	4.34 ± 1.22	t = −19.877	<0.001
Facilitating conditions	3.66 ± 0.18	4.01 ± 1.41	t = −14.760	<0.001
Willingness to use AED	462/652 (70.8%)	547/650 (84.15%)	χ2 = 32.99	0.001
CPR willingness distribution	Likert distribution	Likert distribution	χ2 = 22.995	0.001

The training-associated pattern was consistent with the structural model: the largest ETPB change was observed in perceived behavioral control, which was also one of the strongest predictors of rescue intention in the structural equation model (Table 9).

**Table 9:** Changes in practical CPR performance pass rates before and after first-aid training.

Practical domain	Pre-training pass	Post-training pass	Test statistic	*p*
Scene assessment/calling for help	221 (33.8%)	621 (95.2%)	χ^2^ = 177.570	<0.001
Chest compressions	160 (24.5%)	610 (93.5%)	χ^2^ = 81.566	<0.001
Airway opening	133 (20.3%)	565 (86.6%)	χ^2^ = 11.570	<0.001
Rescue breathing	256 (39.2%)	650 (99.6%)	χ^2^ = 311.741	0.049
Reassessment	23 (3.5%)	559 (85.7%)	χ^2^ = 46.366	0.006
Compression-to-ventilation ratio	231 (35.4%)	543 (83.2%)	χ^2^ = 59.216	0.031

## 4 Discussion

### 4.1 Principal findings and contribution

This theory-driven study examined CPR rescue intention among adults in Hubei Province using an ETPB framework. The central finding was that CPR rescue intention was shaped more strongly by perceived behavioral control and facilitating conditions than by behavioral attitude alone. Many respondents recognized the value of bystander rescue, yet willingness to perform CPR depended mainly on whether they believed they could act correctly and whether training, resources, and supportive conditions were accessible.

This study extends previously published AED-focused descriptive evidence by shifting the research question from AED knowledge and PAD willingness to the psychological and contextual determinants of CPR rescue intention. This distinction is important because the ETPB items reported in the present manuscript primarily measured CPR-related rescue intention, while AED-related findings were retained as contextual background and part of the training evaluation.

### 4.2 Interpretation of the ETPB model

Perceived behavioral control and facilitating conditions were the strongest predictors of CPR rescue intention. This suggests that bystander rescue willingness is limited primarily by self-efficacy and enabling resources. A person may believe that saving lives is morally valuable and socially approved, but still hesitate when confidence, training access, legal clarity, or practical support is insufficient.

The non-significant path from implementation context should be interpreted cautiously. One explanation is that contextual concerns such as legal liability, disease exposure, public attention, and scene safety may influence intention indirectly through perceived control and facilitating conditions (12–15). Another explanation is that the current implementation-context items may not fully capture the complexity of real emergency situations. Future studies should refine this domain and examine potential mediation or moderation effects.

### 4.3 Psychometric implications

The ETPB-based questionnaire showed acceptable preliminary reliability and validity. Cronbach alpha, KMO, Bartlett test, CR, AVE, and factor loadings supported the internal consistency and convergent validity of the instrument. However, EFA and CFA were conducted on the same dataset; therefore, the factor structure should be considered preliminary and requires replication in independent samples.

Providing item-level descriptive statistics and factor loadings improves transparency and allows readers to assess whether the instrument measures psychological and contextual determinants of CPR rescue intention beyond simple knowledge or awareness.

### 4.4 Training-associated changes

The supplementary training evaluation provided practical support for the ETPB interpretation. The largest domain-level change was observed in perceived behavioral control, which was also one of the strongest predictors in the structural model. This suggests that standardized first-aid training may influence rescue intention not only through knowledge acquisition but also through confidence building and perceived ability to act.

Non-technical training components are important. Clarifying legal and safety concerns, reducing fear of harming the victim, increasing repeated hands-on practice, and providing clear learning pathways may be essential for translating positive attitudes into willingness to act. Nevertheless, because the training evaluation used a single-group pre-post design, the observed changes may reflect training, repeated testing, self-selection, or unmeasured factors. Controlled or randomized studies are needed to determine causal effectiveness (16–18).

### 4.5 Importance of previous CPR/AED training experience

Previous CPR/AED training experience is important for interpreting CPR rescue intention. In this study, 26.3% of respondents reported previous CPR/AED training. The supplementary multi-group SEM and path-by-path critical-ratio comparison further showed that training history moderated two specific ETPB pathways. Specifically, the pathway from perceived behavioral control to CPR rescue intention and the pathway from facilitating conditions to CPR rescue intention differed significantly between trained and untrained participants. Among trained participants, perceived behavioral control had a significantly stronger association with CPR rescue intention. In contrast, among untrained participants, facilitating conditions had a significantly stronger association with CPR rescue intention.

This finding provides meaningful public health implications. For individuals who have received CPR/AED training, willingness to perform CPR may depend more strongly on whether they believe they can correctly judge cardiac arrest, call for emergency help, and perform chest compressions. Therefore, promotion strategies for trained laypeople should not only provide knowledge, but should also reinforce perceived competence through refresher training, simulation-based practice, feedback on compression quality, scenario rehearsal, and confidence-building interventions. These strategies may help trained individuals translate previous training experience into actual readiness to act.

In contrast, for individuals without previous CPR/AED training, facilitating conditions played a stronger role in shaping CPR rescue intention. This suggests that untrained laypeople may be limited less by negative attitudes than by insufficient access to training opportunities, unclear learning pathways, limited organizational support, and uncertainty about legal or safety issues. Public health strategies for untrained individuals should therefore prioritize low-threshold CPR/AED education, community-based training access, online introductory learning, visible AED and CPR learning resources, legal and safety clarification, and supportive social environments. These findings are consistent with Dainty et al. (11), who emphasized the importance of understanding lay responder experience in out-of-hospital cardiac arrest.

### 4.6 Public health implications

These findings suggest that CPR education should move beyond information dissemination. Public health programs should prioritize low-threshold practice opportunities, modular refresher courses, community-based simulation, accessible AED training, legal and safety education, and repeated confidence-building exercises. Because facilitating conditions were strongly associated with rescue intention, improving the convenience and availability of training may be as important as improving individual motivation (19, 20).

The results also suggest that public campaigns should explicitly address legal concerns, fear of harm, and lack of confidence, which have also been reported in studies from China and other countries. Similar barriers have been reported in Chinese online-trained populations and in surveys from Taiwan and Japan, where fear of performing CPR incorrectly, fear of harming the victim, and concerns about legal or social consequences reduced willingness to intervene (6, 14, 15). Education strategies should therefore combine technical competence, psychological readiness, and supportive community environments.

### 4.7 Limitations

This study has several limitations. First, convenience, snowball, and public-place sampling may limit representativeness. The sample included relatively high proportions of college-educated participants, urban residents, and participants with medical-related occupations, so the findings may not fully represent the broader adult population of Hubei Province, especially rural residents and those with lower educational attainment. Second, knowledge analyses may be affected by skip logic because respondents reporting complete unfamiliarity did not complete subsequent knowledge items. This may have introduced selection bias and overestimated public knowledge levels. Third, the cross-sectional design precludes causal inference for the structural model. Fourth, EFA and CFA were conducted using the same dataset, which may have overestimated model stability. Fifth, although supplementary multi-group SEM and path-by-path critical-ratio comparison were conducted to identify training-related moderated pathways, the results should still be interpreted cautiously. Previous CPR/AED training experience was self-reported and was measured as a binary variable, without further distinction by training type, training quality, time since last training, refresher training, or actual skill retention. In addition, a full measurement invariance testing sequence was not conducted. Future studies should collect more detailed training-related information and further validate the moderated pathways using independent samples and full measurement invariance testing.

## 5 Conclusion

This revised manuscript positions the Hubei project as a theory-driven investigation of CPR rescue intention. CPR rescue intention was shaped primarily by perceived behavioral control and facilitating conditions rather than by attitude alone. The ETPB-based measurement model showed acceptable preliminary psychometric performance, and the structural model identified modifiable targets for CPR education. The supplementary training evaluation suggested training-associated changes in CPR/AED knowledge, practical performance, confidence, willingness, and key ETPB domains, especially perceived behavioral control. The supplementary multi-group SEM and path-by-path critical-ratio comparison further showed that previous CPR/AED training experience moderated two key ETPB pathways. Perceived behavioral control played a stronger role among trained laypeople, whereas facilitating conditions played a stronger role among untrained laypeople. These findings highlight the importance of considering training background when interpreting lay responders’ psychological and contextual readiness for CPR and suggest that CPR promotion strategies should be tailored to different training histories. Future studies should validate these findings using independent samples, longitudinal designs, full measurement invariance testing, and more detailed measures of CPR/AED training type, recency, quality, and skill retention.

## Data Availability

The raw data supporting the conclusions of this article will be made available by the authors, without undue reservation.
